# *Aeromonas* species obtained from different farmed aquatic species in India and Taiwan show high phenotypic relatedness despite species diversity

**DOI:** 10.1186/s13104-021-05716-3

**Published:** 2021-08-16

**Authors:** Saurabh Dubey, Biswajit Maiti, Shivani Kallappa Girisha, Rakesh Das, Mustapha Lamkhannat, Stephen Mutoloki, Shih-Chu Chen, Indrani Karunasagar, Øystein Evensen, Hetron M. Munang´andu

**Affiliations:** 1 Faculty of Veterinary Medicine, Norwegian University of Life Sciences, P.O. Box 369, 0102 Oslo, Norway; 2 UNESCO MIRCEN for Medical and Marine Biotechnology, Nitte University Centre for Science Education and Research, NITTE (Deemed to be University), Paneer Campus, Deralakatte, Mangalore, 575018 India; 3 Department of Fisheries Microbiology, Karnataka Veterinary, Animal & Fisheries Sciences University, College of Fisheries, Mangalore, 575002 India; 4 ICAR-Central Institute of Freshwater Aquaculture (CIFA), Kausalyaganga, Odisha 751002 India; 5 Department of Veterinary Medicine, National Pingtung University of Science and Technology, Pingtung, Taiwan

**Keywords:** *Aeromonas hydrophila*, *A*. *sobria*, *A*. *veronii*, 16S rRNA, *gyrB*, Phenotype, Genotype

## Abstract

**Objectives:**

Aeromonads cause severe diseases in farmed aquatic organisms. Herein, we examined 28 isolates causing disease in farmed aquatic organisms from India (n = 24) and Taiwan (n = 4) to gain insight of their genotypic and phenotypic properties.

**Results:**

API 20NE biochemical phenotyping showed ≥ 90% similarity classifying all isolates as *Aeromonas hydrophila*. 16S rRNA genotyping showed ≥ 98% homology among all isolates with *A. sobria* (NR119044.1ATCC), *A*. *veronii* (MK990549.1), *A. caviae* (NR029252.1) and *A. hydrophila* (MG984625.1ATCC) and other reference strains. In contrast, *gyrB* showed a higher intraspecies diversity (≥ 96%) than 16S rRNA delineating the 28 isolates into three groups. Group-I consisted of seven Indian isolates clustered with *A. sobria* (MK484163.1ATCC), group-II comprised of five Indian and two Taiwanese isolates clustered with *A. veronii* AF417626.1ATCC while group-III had 11 Indian and three Taiwanese isolates grouped with *A. hydrophila* (AY987520.1 and DQ519366.1) reference strains. None of our isolates clustered with *A. caviae* (AJ868400.1ATCC) reference strain. These findings suggest that *A. sobria*, *A. veronii* and *A. hydrophila* could be the etiological agents of diseases observed in farmed fish and soft-shelled turtles (*Pelodiscus sinensis*) examined in this study. Overall, our findings accentuate the importance of combining phenotyping with genotyping for correct taxonomic classification of *Aeromonas* spp. in Aquaculture.

**Supplementary Information:**

The online version contains supplementary material available at 10.1186/s13104-021-05716-3.

## Introduction

Aeromonads cause diseases characterized by severe hemorrhages and septicemia in farmed aquatic organisms [1]. Identification of *Aeromonas* species infecting aquatic organisms call for phenotype and genotype characterization. While phenotyping based on biochemical tests is widely used for bacteria characterization, it sometimes produces conflicting results due to extreme diversity between and within species rendering genotyping to be a better option [1]. Although 16S rRNA is the most widely used molecular marker for genotyping due to its reliability in determining inter- and intragenic genealogical interrelationships between bacteria species [2], its variable regions vary in size and organization resulting in poor intraspecies resolution. Thus, housekeeping genes like *gyrB* with a mean synonymous substitution rate four times faster than 16S rRNA are more reliable for intraspecies genotyping than 16S rRNA [3]. Herein, we wanted to identify *Aeromonas* species causing diseases in farmed organisms in India and Taiwan using *gyrB* and 16S rRNA, and API 20 NE biochemical characterization.

## Main text

### Material and methods

#### Sample collections

Fish and soft-shelled turtles submitted to Aquatic Animal Health Centers in India and Taiwan accompanied by clinical and pathology reports were used in this study. Of the 24 samples from India, samples from North India were from *Cyrprinus carpio*, *Clarias batrachus* and *Oreochromis niloticus* collected from eight different farms while samples from South India were from *Labeo rohita*, *Catla catla*, *Cirrhinus mrigala*, and *Carassius auratus* collected from 20 different farms. Samples from Taiwan were from *Hyperprosopon ellipticum*, *O. niloticus*, and *Pelodiscus sinensis* collected from six different farms. Swabs from internal organs such as kidney, muscle, liver, and heart were used for bacteria isolation in trypticase soy agar (TSA) and trypticase soy broth (TSB).

#### Bacterial isolation

A total of 33 isolates obtained from India and Taiwan (Additional file 1: Table S1) were initially cultured on TSA and TSB for bacteria isolation before culture on Aeromonas isolation agar (AIA) and Rimler Shotts (RS) selective medium (Sigma-Aldrich, USA). Characteristic single green colonies from AIA and yellow colonies from RS medium were streaked on TSA for pure colony isolation.

#### Phenotypic characterization

Morphology examination was done after Gram staining by microscopy. All isolates were cultured on 5% sheep blood agar (SBA) for hemolysis examination. Biochemical tests were done using the API20 NE kit (BioMérieux, Marcy l'Etoile, France).

#### Genotype characterization

Bacteria genomic DNA was extracted as described [4]. PCR was performed using the AccuStart *Taq* DNA Polymerase HiFi (Quanta, Biosciences) using 16S rRNA and *gyrB* primers (Additional file 2: Table S2) as previous described [5]. Purified PCR products were sequenced on commercial basis by GATC-Biotech (GATC-Biotech, Germany). Sequences were used to generate 16S rRNA and *gyrB* phylogenetic trees in MEGA7 bioinformatics software [6]. The evolutionary history for each tree was inferred using the Maximum Composite Likelihood method [7] as described in our previous study [8]. Genetic distances were computed using Kimura’s two-parameter value [7].

### Results

#### Clinical and gross pathology observations

Clinical signs were characterized by lethargy, poor swimming behavior and reduced feed intake. Pathology was characterized by different conditions such as hemorrhages, ulceration, and fin rot in fish (Additional file 5: Figure S1). High mortalities were reported on fish and soft-shelled turtle farms.

#### Phenotypic characterization of Indian and Taiwanese isolates

Morphological, hemolysis, motility and biochemical results are shown in Additional file 1: Table S1. Of the 33 isolates that produced colonies on TSA, only 28 isolates (84.84%, n = 33) grew on AIA and RS selective media exhibiting characteristic of green and yellow colonies, respectively (Additional file 1: Table S1, Additional file 6: Figure S2 (1, 2)). In addition, the 28 isolates (84.84%, n = 33) showed β-hemolysis while five (15.15%, n = 33) had no hemolytic zones on 5% SBA (Additional file 6: Figure S2 (3)). Microscopic examination showed Gram-negative vibrio shaped bacteria characteristic of *Aeromonas* spp.

Of the 33 isolates examined using the API-20NE kit, 28 isolates showed characteristic properties having an overall score of 6,566,654 leading to classification of these isolates as *A. hydrophila* [9]. Despite so, phenotypic similarities and differences were observed among the 28 isolates classified as *A. hydrophila* based on the API-20NE 21-biochemical tests. All 28 isolates were positive for 15 and negative for four tests giving a similarity of 90.48% (n = 21 API-20NE tests) (Table 1). Major differences between isolates were based on d-arabinose (ARA) and malic acid (MLT) and were classified into four major categories based on ARA/MLT (−/+, +/−, +/+, −/−) utilization (Additional file 3: Table S3). These results are summarized in Additional file 3: Table S3, which shows that isolates from two species *L. rohita* and *C. carpio* (India) had a −/+ ARA/MLT utilization, with only one isolate from India being positive (+/+) for both sugars. All isolates from *C. catla*, *C. batrachus*, and *C. mrigala* had +/− ARA/MLT utilization. The Taiwanese *H. ellipticum* and most of the *O. niloticus* isolates from India were positive for both ARA/MLT (+/+). On the other hand, *P. sinensis* and *C. auratus* isolates were ARA/MLT negative (−/−). In summary, these observations suggest that utilization of these sugars could be influenced by host species adaption.

**Table 1:**
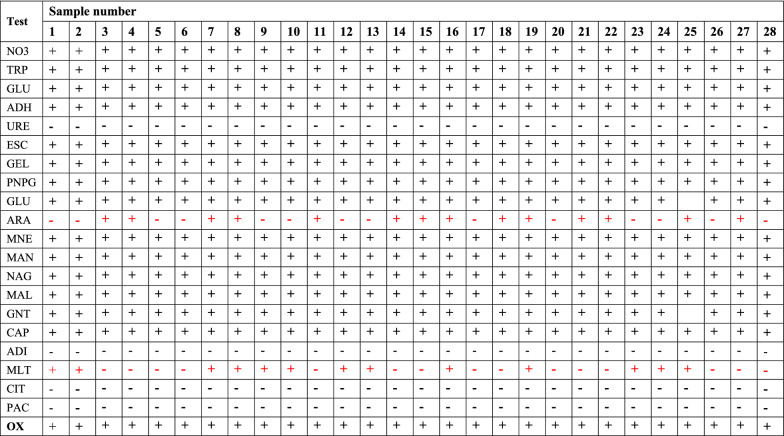
API-20 NE characterization of *Aeromonas hydrophila* isolates from India and Taiwan

The bold values indicate the similarity while the red shows differences between isolates

API 20 NE result: identification: (+) positive; (−) negative, full form of all the test are NO_3_ (potassium nitrate), TRP (l-tryptophane), GLU (d-glucose), ADH (l-arginine), URE (urea), ESC (Esculin ferric citrate), GEL (Gelatin), PNPG (4-nitrophenyl-β-d-galactopyranoside), GLU (d-glucose), ARA (l-arabinose), MNE (d-mannose), MAN (d-mannitol), NAG (*N*-acetyl-glucosamine), MAL (d-maltose), GNT (potassium gluconate), CAP (capric acid), ADI (adipic acid), MLT (malic acid), CIT (trisodium citrate), PAC (phenylacetic acid) and OX (oxidase test)

#### Genotype characterization based on 16S rRNA and *gyrB* genes

The 16S rRNA and *gyrB* PCR products generated 840 bp and 580 bp amplicons from all isolates, respectively (Additional file 4: Table S4).

##### Phylogenetic analysis of 16S rRNA

**Fig. 1.**
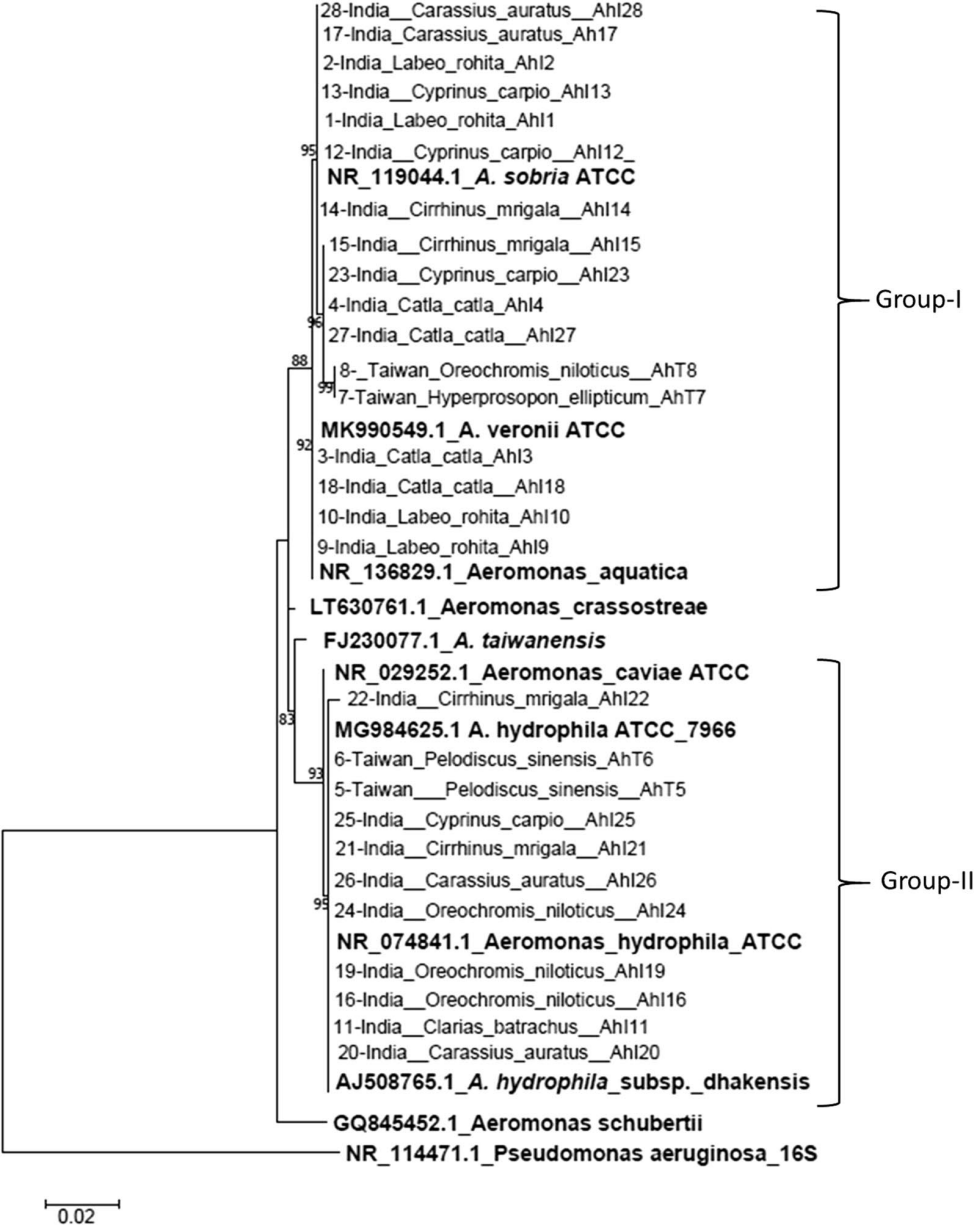
16S rRNA phylogenetic tree. The overall genetic distance (nucleotide identity) divergence for all 28 isolates varied between 0.000 and 0.020 (98.0–100% similarity) while divergence of our isolates with *A. schuberti* was 0.024 (2.4%). The genetic distance divergence between our isolates and *Pseudomonas aeruginosa* used as an out group was estimated at 0.153 (15.3%)

Of the 33 isolates obtained from TSA, three were characterized as *Enterobacter cloacae* while two were characterized as *Acinobacter* spp. using 16S rRNA sequencing. The remaining 28 isolates had ≥ 98% nucleotide identity similarities (E-value = 0.0) with *A. hydrophila* MG984625.1ATCC, *A. hydrophila*_subsp_dhakensis AJ508765.1, *A. caviae* NR_029252.1ATCC, *A. sobria* NR_119044.1ATCC, *A. aquatica* NR_136829.1, *A. crassostreae* LT630761.1, *A. taiwanensis* FJ230077.1, and *A. veronii* MK990549.1ATCC reference strains (Fig. 1). The 16S rRNA phylogenetic tree put all 28 isolates in two groups of which 17 were clustered with *A. sobria* NR_119044.1ATCC, *A. veronii* MK990549.1 ATCC and *A. aquatica* NR_136829.1 reference strains. The remaining 10 isolates were clustered with *A. hydrophila* (MG984625.1ATCC and NR_074841.1ATCC), *A. hydrophila*_subsp_dhakensis AJ508765.1 and *A. caviae* (NR_029252.1 ATCC) reference strains that included two Taiwanese *P. sinensis*. Note that the 16S rRNA tree put *A. hydrophila* (NR_074841.1ATCC and MG984625.1) and *A. hydrophila*_subsp_dhakensis AJ508765.1 as highly homologous with *A. caviae* NR_029252.1ATCC while the *A. crassostreae* (LT630761.1) and *A. taiwanensis* (FJ230077.1) reference strains were in between groups I and II (Fig. 1). All isolates were distantly related with *A. schubertii* (CQ845452.1) with a nucleotide identity disparity of 2.4% and further separated from the *Pseudomonas euroginosa* (NR_114471.1) outgroup with a nucleotide identity disparity of 15%.

##### Phylogenetic analysis of *gyrB* gene

**Fig. 2:**
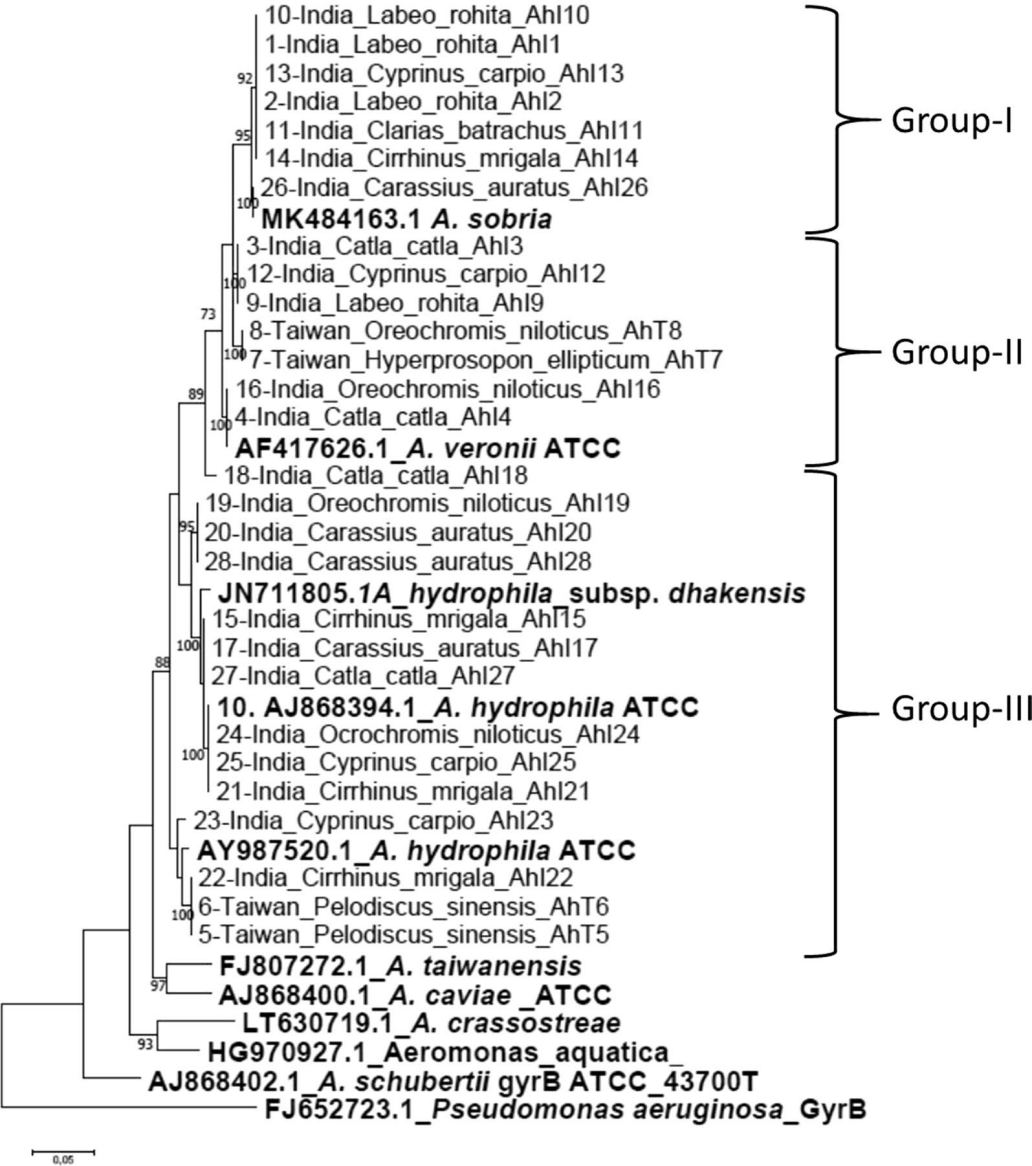
*gyrB* phylogenetic tree. The overall genetic distance (nucleotide identity) divergence for all fish and soft-shelled turtle isolates (*Pelodiscus sinensis*) from India and Taiwan varied between 0.000 and 0.047 (95.7–100% similarity). The genetic distance divergence between our isolates and *A. schuberti* was 0.130 (13.0%). The variability between our isolates and *P. euroginosa* used as an outgroup were estimated at 0.304 (30.4%)

Of the 33 isolates examined, only 28 isolates that were positive for AIA and RS growth on selective media produced *gyrB* sequences. The *gyrB* tree put the 28 isolates in three major groups (Fig. 2). Group I consisted of seven Indian isolates put close to the *A. sobria* MK484163.1 reference strain while Group II comprised of five Indian and two Taiwanese isolates (*H. ellipticum* and *O. niloticus*) clustered with *A. veronii* AF417626.1ATCC. Group-III consisted of 10 Indian isolates grouped with *A. hydrophila* AJ868394.1ATCC and *A. hydrophila*_subsp. dhakensis JN11805.1A while four isolates comprising of two Taiwanese *P. sinensis* and Indian isolates grouped with the *A. hydrophila* AY987520.1ATCC reference strains. We found a nucleotide identity similarity of ≥ 94.0% among all isolates together with all *Aeromonas* reference strains. Contrary to the 16S rRNA phylogenetic tree, the *gyrB* tree shows that all isolates were distantly related with the *A. caviae* (AJ868400.1ATCC), *A. taiwanensis* (FJ807272.1), *A. aquatica* (HG970927.1) and *A. crassostreae* (LT630719.1). Similarly, all isolates were distantly related with *A. schubertii* (AJ868402.1ATCC) and *P. euroginosa* (FJ652723.1ATCC) outgroup with nucleotide identity disparity of 13% and 30.4%, respectively.

### Discussion

The main finding from this study is that farmed fish and soft-shelled turtle from India and Taiwan were infected by different *Aeromonas* species. Clinical signs of lethargy and poor swimming behavior, and pathology characterized by hemorrhages, fin loss and tail rot seen in this study are in line with previous reports [10–12]. For the soft-shelled turtle, previous studies show reduced growth rate, softening of the dorsal shell and acute mortalities [13, 14]. Altogether, these observations show that *Aeromonas* species cause disease in a wide host range in aquaculture.

Growth on selective media, motility, β-hemolysis and morphological properties showed that only 28 out of 33 isolates examined produced phenotypic traits characteristic of *A. hydrophila* [15–17]. All isolates classified as *A. hydrophila* by API-20NE were positive for 15 reactions that included oxidase and indole; glucose, maltose, and mannose fermentation; nitrate reduction; arginine, d-mannitol, *N*-acetyl-glucosamine and d-maltose hydrolysis; gas and acetoin production from glucose; and, lysine decarboxylation in line with other scientists who found similar properties in fish isolates [18, 19]. In addition, all isolates were negative for urea, esculin ferric citrate, trisodium citrate and phenylacetic acid being in line with Martin et al. [19] who reported similar findings from fish isolates. However, differences in arabinose and malic acid reactions grouped the 28 isolates into four major groups based on species of origin (Additional file 3: Table S3) pointing to possibilities of host species adaption in different environments. Despite so, biochemical analysis show that the 28 strains had a high phenotypic similarity (90%, n = 21 biochemical reactions).

In line with previous studies showing that the genus *Aeromonas* is one of the most tightly defined genera because of high 16S rRNA intra-species similarities (97–100%) [20, 21], our findings show a high similarity (≥ 98%) among *A. caviae* (NR_029252.1ATCC), *A. sobria* (NR_119044.1ATCC) *A. veronii* (MK990549.1ATCC), *A. crassostreae* (LT630761.1), *A. taiwanensis* (FJ230077.1) *A. hydrophila*_subsp_dhakensis (AJ508765.1) and *A. hydrophila* (NR_074841.1ATCC and MG984625.1 ATCC) reference strains. We also found a high intra-species similarity (≥ 98%) among the 28 isolates from nine different aquatic organisms from India and Taiwan suggesting that our isolates were closely related with the *A. hydrophila*, *A. caviae*, *A. sobria* and *A. veronii* reference strains based on 16S rRNA classification. On the contrary, the *gyrB* tree showed a higher disparity (≤ 6.0%) among the reference strains than the 16S rRNA tree (≤ 2.0%) as shown that it delineated isolates homologous with the *A. sobria* (MK484163.1), *A. veronii* (AF417626.1ATCC), and *A. hydrophila* (AJ868394.1ATCC) reference strains into separate clusters. Our findings also show that the *gyrB* tree separated group I isolates clustered with *A. sobria* (MK484163.1) from group-II isolates clustered with *A. veronii* (AF417626.1ATCC). It also separated groups I and II isolates from group-III isolates that were grouped with *A. hydrophila* subsp_<<<dhakensis (JN711805.1A) and *A. hydrophila* reference strains (AY987520.1ATCC and AJ868394.1ATCC) indicating that *A. veronii* and *A. sobria* isolates were different from *A. hydrophila* isolates. In addition, the *gyrB* tree clearly separated our isolates were from *A. caviae* (AJ868400.1ATCC), *A. taiwanensis* (FJ807272.1), *A. crassostreae* (LT630719.1) and *A. aquatica* (HG970927.1) reference strains indicating that none our isolates belonged to these species unlike 16S rRNA, which showed that our isolates were homologous with these reference strains. We also found a high disparity between our *Aeromonas* spp. isolates and *A. schuberti* (AJ868402.1ATCC) with a five times higher disparity in the *gyrB* tree (13.0%) than the 16S rRNA tree (2.4%). Altogether these findings show that *gyrB* has a higher intraspecies differentiation capacity than 16S rRNA. As for interspecies differentiation, the *gyrB* tree (30.4%) showed a higher differentiation capacity of *Aeromonas* spp. from *P. euroginosa* than the 16S rRNA tree (15.0%). In summary, these findings are in line with previous studies that show that *gyrB* has a higher intra- and interspecies differentiation capacity than 16S rRNA [5, 22–24].

Our findings show that farmed fish and soft-shelled turtle from India and Taiwan were infected by different *Aeromonas* species. Phenotyping based on API 20NE showed a high similarity (> 90%, n = 28) with all isolates classified as *A. hydrophila.* Genotyping showed species diversity of which *gyr*B phylogenetic analysis gave better intra- and interspecies differentiation than 16S rRNA.

## Limitations

Major limitations are that we could not to determine whether differences in arabinose and malic acid reactions that grouped the 28 isolates in four groups based on species of origin (Additional file 3: Table S3) is influenced by environmental host species adaption due to sample size limitation. Genotyping based on two genes (16S rRNA and *gyr*B) could be a limiting factor for intraspecies differentiation for large sample sizes, but multi-loci sequence types (MLST) incorporating several housekeeping genes might have a higher resolution. Future studies should use large sample sizes and include several Reference species.

## Supplementary Information

**Additional file 1: Table S1.** Characterization of isolates based on growth media, catalase test, hemolysis and motility.

**Additional file 2: Table S2.** Primer sequences for 16S rRNA and *gyrB*, genes.

**Additional file 3: Table S3.** API 20 NE characterization of *Aeromonas hydrophila* isolates based on l-arabinose and malic acid utilization.

**Additional file 4: Table S4.** Host species, country of origin and GenBank accession numbers of *Aeromonas hydrophila*.

**Additional file 5: Figure S1.** Fish infected by *Aeromonas* species. **A** Hemorrhages on body surfaces including the tail, eyes, mouth, gill operculum and fins in goldfish. **B** Hemorrhages on body surfaces and fin in (rohu) *Labeo rohita*. **C**, **D** Loss of fins in rohu (*L. rohita*). **E** Hemorrhages in lower abdomen in rohu (*L. rohita*). **F** Hemorrhages on the fins of *Clarias batrachus*.

**Additional file 6: Figure S2.***Aeromonas* colonies on Aeromonas isolation agar (AIA), Rimler Shotts (RS) agar and 5% sheep blood agar (5% SBA). **Figure S2.** (1) *Aeromonas* spp. colonies showing characteristic green color on AIA agar. (2) *Aeromonas* spp*.* colonies showing yellow colonies on RS agar while, (3) shows *Aeromonas* spp. on 5% sheep blood agar (5% BSA) exhibiting β-hemolysis zones around the colonies.

## Data Availability

Data used for genotyping is shown in Additional file 2: Table S2 (Accession numbers for NMBI).

## Abbreviations

16 rRNA: 16S ribosomal ribonucleic acid
gyrB: DNA gyrase subunit B
MLST: Multilocus sequence typing
API 20NE: Analytical profile index 20 non-enterobacteriaceae
ATCC: American type cell collection
PCR: Polymerase chain reaction
ARA: d-Arabinose
MLT: Malic acid
AIA: Aeromonas isolation agar
RS: Rimler Shotts
TSA: Trypticase soy agar
TSB: Trypticase soy broth
SBA: Sheep blood agar
