# Supplementary material for: Aeromonas species obtained from different farmed aquatic species in India and Taiwan show high phenotypic relatedness despite species diversity
Source: BMC Res Notes. 2021 Aug 16;14:313. doi: 10.1186/s13104-021-05716-3 (PMC8365956; doi:10.1186/s13104-021-05716-3)
Supplement: Supplementary file 1 — Additional file 1: Table S1. Characterization of isolates based on growth media, catalase test, hemolysis and motility. [file 13104_2021_5716_MOESM1_ESM.pdf]

Table S1. Characterization of isolates based on growth media, catalase test, hemolysis and motility

No	Source	ID code	Origin	Gram stain	Motility	Growth Media				
						TSA	AIA	RS	5% SBA	β-H/C
**1**	*Labeo rohita*	AhI1	S. India	Gram –ve	+	+	+	+	+	+
**2**	*Labeo rohita*	AhI2	S. India	Gram –ve	+	+	+	+	+	+
**3**	*Catla catla*	AhI3	S. India	Gram –ve	+	+	+	+	+	+
**4**	*Catla catla*	AhI4	S. India	Gram –ve	+	+	+	+	+	+
**5**	*Pelodiscus sinensis*	AhT5	Taiwan	Gram –ve	+	+	+	+	+	+
**6**	*Pelodiscus sinensis*	AhT6	Taiwan	Gram –ve	+	+	+	+	+	+
**7**	*Hyperprosopon ellipticum*	AhT7	Taiwan	Gram –ve	+	+	+	+	+	+
**8**	*Oreochromis niloticus*	AhT8	Taiwan	Gram –ve	+	+	+	+	+	+
**9**	*Oreochromis niloticus*	AhT9	Taiwan	Gram –ve	+	+	-	-	+	-
**10**	*Hyperprosopon ellipticum*	AhT10	Taiwan	Gram –ve	+	+	-	+	+	-
**11**	*Labeo rohita*	AhI11	S. India	Gram –ve	+	+	+	+	+	+
**12**	*Labeo rohita*	AhI12	S. India	Gram –ve	+	+	+	+	+	+
**13**	*Clarias batrachus*	AhI13	N. India	Gram –ve	+	+	+	+	+	+
**14**	*Cyprinus carpio*	AhI14	N. India	Gram –ve	+	+	+	+	+	+
**15**	*Cyprinus carpio*	AhI15	N. India	Gram –ve	+	+	+	+	+	+
**16**	*Cirrhinus mrigala*	AhI16	S. India	Gram –ve	+	+	+	+	+	+
**17**	*Cirrhinus mrigala*	AhI17	S. India	Gram –ve	+	+	+	+	+	+
**18**	*Oreochromis niloticus*	AhI18	N. India	Gram –ve	+	+	+	+	+	+
**19**	*Carassius auratus*	AhI19	S. India	Gram –ve	+	+	+	+	+	-
**20**	*Catla catla*	AhI20	S. India	Gram –ve	+	+	+	+	+	+
**21**	*Oreochromis niloticus*	AhI21	N. India	Gram –ve	+	+	+	+	+	-
**22**	*Carassius auratus*	AhI22	S. India	Gram –ve	+	+	+	+	+	+
**23**	*Cirrhinus mrigala*	AhI23	S. India	Gram –ve	+	+	+	+	+	+
**24**	*Cirrhinus mrigala*	AhI24	S. India	Gram –ve	+	+	+	+	+	+
**25**	*Cyprinus carpio*	AhI25	N. India	Gram –ve	+	+	+	+	+	+
**26**	*Oreochromis niloticus*	AhI26	N. India	Gram –ve	+	+	+	+	+	+
**27**	*Cyprinus carpio*	AhI27	N. India	Gram –ve	+	+	+	+	+	+
**28**	*Carassius auratus*	AhI28	S. India	Gram –ve	+	+	+	+	+	+
**29**	*Catla catla*	AhI29	S. India	Gram –ve	+	+	-	+	+	-
**30**	*Carassius auratus*	AhI30	S. India	Gram –ve	+	+	-	-	+	-
**31**	*Catla catla*	AhI31	S. India	Gram –ve	+	+	-	+	+	-
**32**	*Labeo rohita*	AhI32	S. India	Gram –ve	+	+	-	+	+	-
**33**	*Carassius auratus*	AhI33	S. India	Gram –ve	+	+	-	-	+	-

Note: Abbreviation: N. India= North India, S. India= South India.
