# Supplementary material for: Aeromonas species obtained from different farmed aquatic species in India and Taiwan show high phenotypic relatedness despite species diversity
Source: BMC Res Notes. 2021 Aug 16;14:313. doi: 10.1186/s13104-021-05716-3 (PMC8365956; doi:10.1186/s13104-021-05716-3)
Supplement: Supplementary file 2 — Additional file 2: Table S2. Primer sequences for 16S rRNA and gyrB, genes. [file 13104_2021_5716_MOESM2_ESM.pdf]

Table S2. Primer sequences for 16S rRNA and *gyrB*, genes

Primer name	Direction	Primer	GenBank Acc. No
Ah gyrB	Forward:	TCCGGCGGTCTGCACGGCGT	ATCC_49140
	Reverse:	TTGTCCGGGTTGTACTCGTC	
Ah 16S rRNA	Forward:	AGGGGGATAACAGTTGGA	MG984625.1
	Reverse:	AACGTATTCACCGCAACA	ATCC 7966
