# Supplementary material for: Aeromonas species obtained from different farmed aquatic species in India and Taiwan show high phenotypic relatedness despite species diversity
Source: BMC Res Notes. 2021 Aug 16;14:313. doi: 10.1186/s13104-021-05716-3 (PMC8365956; doi:10.1186/s13104-021-05716-3)
Supplement: Supplementary file 3 — Additional file 3: Table S3. API 20 NE characterization of Aeromonas hydrophila isolates based on l-arabinose and malic acid utilization. [file 13104_2021_5716_MOESM3_ESM.pdf]

Table S3. API 20 NE characterization of *Aeromonas hydrophila* isolates based on L-arabinose and malic acid utilization.

No	Host	Country	ID	ARA	MLT
**1**	*Labeo rohita*	India	1-India_*Labeo rohita*_AhI1	-	+
**2**	*Labeo rohita*	India	2-India_*Labeo rohita*_AhI2	-	+
**9**	*Labeo rohita*	India	9-India_*Labeo rohita*_AhI9	-	+
**10**	*Labeo rohita*	India	10-India_*Labeo rohita*_AhI10	-	+
**12**	*Cyprinus carpio*	India	12-India_ *Cyprinus carpio* _AhI12	-	+
**13**	*Cyprinus carpio*	India	13-India_ *Cyprinus carpio* _AhI13	-	+
**23**	*Cyprinus carpio*	India	23-India_ *Cyprinus carpio* _AhI23	-	+
**25**	*Cyprinus carpio*	India	25-India_ *Cyprinus carpio* _AhI25	+	+
**3**	*Catla catla*	India	3-India_*Catla catla* _AhI3	+	-
**4**	*Catla catla*	India	4-India_*Catla catla* _AhI4	+	-
**18**	*Catla catla*	India	18-India_*Catla catla* _AhI18	+	-
**27**	*Catla catla*	India	27-India_*Catla catla* _AhI27	+	-
**11**	*Clarias batrachus*	India	11-India_ *Clarias batrachus* _AhI11	+	-
**14**	*Cirrhinus mrigala*	India	14-India_ *Cirrhinus mrigala* _AhI14	+	-
**15**	*Cirrhinus mrigala*	India	15-India_ *Cirrhinus mrigala* _AhI15	+	-
**21**	*Cirrhinus mrigala*	India	21-India_ *Cirrhinus mrigala* _AhI21	+	-
**22**	*Cirrhinus mrigala*	India	22-India_ *Cirrhinus mrigala* _AhI22	+	-
**7**	*Hyperprosopon ellipticum*	Taiwan	7- Taiwan _ *Hyperprosopon ellipticum* _AhT7	+	+
**8**	*Oreochromis niloticus*	Taiwan	8- Taiwan _ *Oreochromis niloticus* _AhT8	+	+
**16**	*Oreochromis niloticus*	India	16-India_ *Oreochromis niloticus* _AhI16	+	+
**19**	*Oreochromis niloticus*	India	19-India_ *Oreochromis niloticus* _AhI19	+	+
**24**	*Oreochromis niloticus*	India	24-India_ *Oreochromis niloticus* _AhI24	-	+
**5**	*Pelodiscus sinensis*	Taiwan	5-Taiwan _ *Pelodiscus sinensis* _AhT5	-	-
**6**	*Pelodiscus sinensis*	Taiwan	6- Taiwan _ *Pelodiscus sinensis* _AhT6	-	-
**17**	*Carassius auratus*	India	17-India_ *Carassius auratus* _AhI17	-	-
**20**	*Carassius auratus*	India	20-India_ *Carassius auratus* _AhI20	-	-
**26**	*Carassius auratus*	India	26-India_ *Carassius auratus* _AhI26	-	-
**28**	*Carassius auratus*	India	28-India_ *Carassius auratus* _AhI28	-	-
