# Supplementary material for: Aeromonas species obtained from different farmed aquatic species in India and Taiwan show high phenotypic relatedness despite species diversity
Source: BMC Res Notes. 2021 Aug 16;14:313. doi: 10.1186/s13104-021-05716-3 (PMC8365956; doi:10.1186/s13104-021-05716-3)
Supplement: Supplementary file 4 — Additional file 4: Table S4. Host species, country of origin and GenBank accession numbers of Aeromonas hydrophila. [file 13104_2021_5716_MOESM4_ESM.pdf]

Table S4. Host species, country of origin and GenBank accession numbers of *Aeromonas hydrophila*

No	Host	Country	gyrB	16 S NCBI	ID
**1**	*Labeo rohita*	India	MG193618	MF806600	1-India_*Labeo rohita*_AhI1
**2**	*Labeo rohita*	India	MG050699	MF806601	2-India_*Labeo rohita*_AhI2
**3**	*Catla catla*	India	MG050700	MF806602	3-India_*Catla catla* _AhI3
**4**	*Catla catla*	India	MG193619	MF806603	4-India_*Catla catla* _AhI4
**5**	*Pelodiscus sinensis*	Taiwan	MG050709	MF948884	5-Taiwan _ *Pelodiscus sinensis* _AhT5
**6**	*Pelodiscus sinensis*	Taiwan	MG050710	MF948885	6- Taiwan _ *Pelodiscus sinensis* _AhT6
**7**	*Hyperprosopon ellipticum*	Taiwan	MG050707	MF967406	7- Taiwan _ *Hyperprosopon ellipticum* _AhT7
**8**	*Oreochromis niloticus*	Taiwan	MG050708	MF767520	8- Taiwan _ *Oreochromis niloticus* _AhT8
**9**	*Labeo rohita*	India	MG050701	MF806604	9-India_*Labeo rohita*_AhI9
**10**	*Labeo rohita*	India	MG050702	MF927579	10-India_*Labeo rohita*_AhI10
**11**	*Clarias batrachus*	India	MG193620	MF807157	11-India_ *Clarias batrachus* _AhI11
**12**	*Cyprinus carpio*	India	MG050703	MF927580	12-India_ *Cyprinus carpio* _AhI12
**13**	*Cyprinus carpio*	India	MG050704	MF927581	13-India_ *Cyprinus carpio* _AhI13
**14**	*Cirrhinus mrigala*	India	MG193621	MF927582	14-India_ *Cirrhinus mrigala* _AhI14
**15**	*Cirrhinus mrigala*	India	MG193622	MF927583	15-India_ *Cirrhinus mrigala* _AhI15
**16**	*Oreochromis niloticus*	India	MG193623	MF927584	16-India_ *Oreochromis niloticus* _AhI16
**17**	*Carassius auratus*	India	MG193624	MF928405	17-India_ *Carassius auratus* _AhI17
**18**	*Catla catla*	India	MG193625	MF927585	18-India_*Catla catla* _AhI18
**19**	*Oreochromis niloticus*	India	MG193626	MF928406	19-India_ *Oreochromis niloticus* _AhI19
**20**	*Carassius auratus*	India	MG193627	MF962583	20-India_ *Carassius auratus* _AhI20
**21**	*Cirrhinus mrigala*	India	MG193628	MF948883	21-India_ *Cirrhinus mrigala* _AhI21
**22**	*Cirrhinus mrigala*	India	MG193629	MF942356	22-India_ *Cirrhinus mrigala* _AhI22
**23**	*Cyprinus carpio*	India	MG050730	MF942355	23-India_ *Cyprinus carpio* _AhI23
**24**	*Oreochromis niloticus*	India	MG193631	MF948882	24-India_ *Oreochromis niloticus* _AhI24
**25**	*Cyprinus carpio*	India	MG050706	MF942355	25-India_ *Cyprinus carpio* _AhI25
**26**	*Carassius auratus*	India	MG193632	MF942129	26-India_ *Carassius auratus* _AhI26
**27**	*Catla catla*	India	MG193633	MF943216	27-India_*Catla catla* _AhI27
**28**	*Carassius auratus*	India	MG050705	MF942128	28-India_ *Carassius auratus* _AhI28
