# Supplementary figures and images for: Aeromonas species obtained from different farmed aquatic species in India and Taiwan show high phenotypic relatedness despite species diversity
Source: BMC Res Notes. 2021 Aug 16;14:313. doi: 10.1186/s13104-021-05716-3 (PMC8365956; doi:10.1186/s13104-021-05716-3)

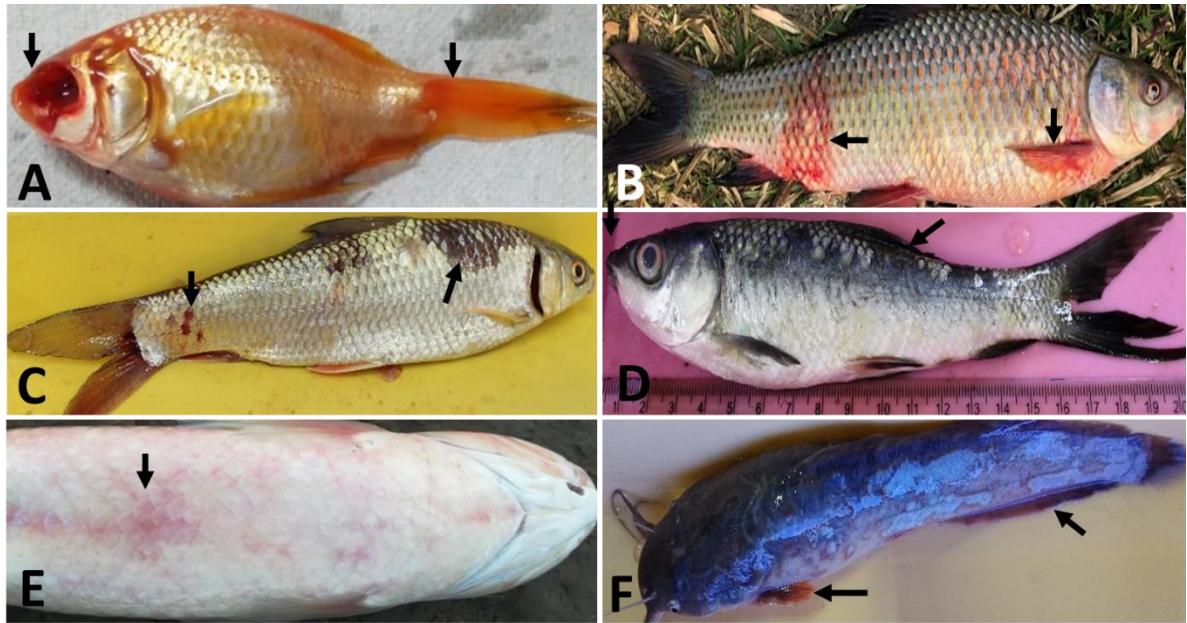

Figure S1

Supplement: Supplementary file 5 — Additional file 5: Figure S1. Fish infected by Aeromonas species. A Hemorrhages on body surfaces including the tail, eyes, mouth, gill operculum and fins in goldfish. B Hemorrhages on body surfaces and fin in (rohu) Labeo rohita. C, D Loss of fins in rohu (L. rohita). E Hemorrhages in lower abdomen in rohu (L. rohita). F Hemorrhages on the fins of Clarias batrachus. [file 13104_2021_5716_MOESM5_ESM.pdf]

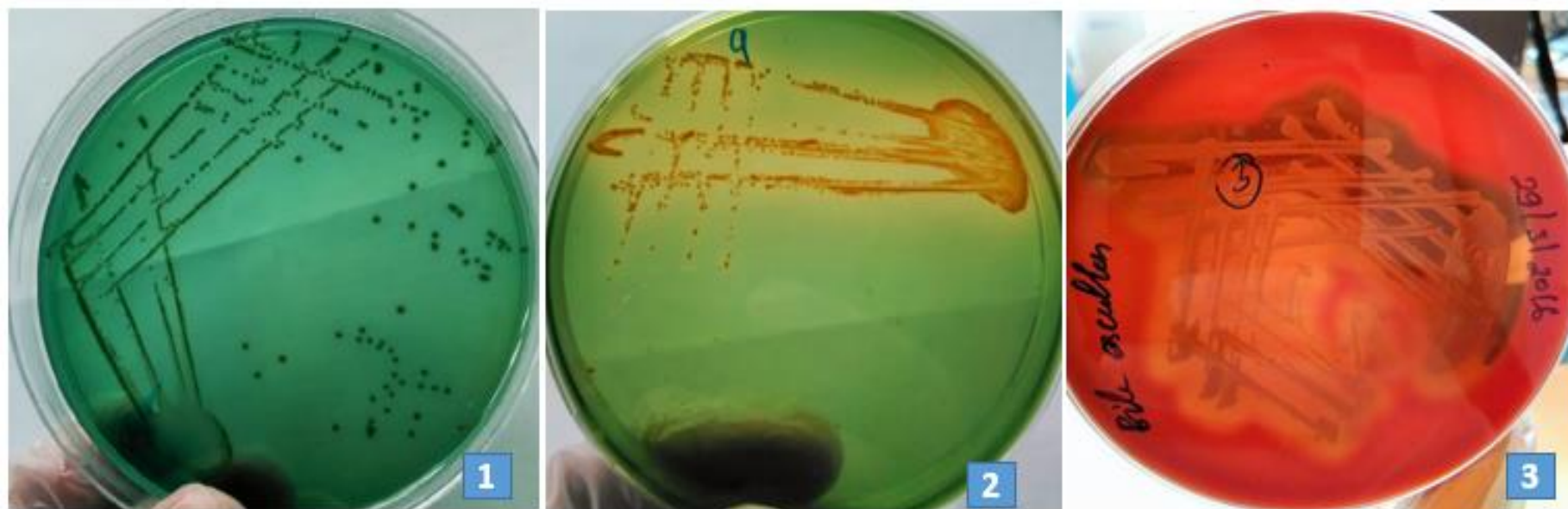

Figure S2

Supplement: Supplementary file 6 — Additional file 6: Figure S2.Aeromonas colonies on Aeromonas isolation agar (AIA), Rimler Shotts (RS) agar and 5% sheep blood agar (5% SBA). Figure S2. (1) Aeromonas spp. colonies showing characteristic green color on AIA agar. (2) Aeromonas spp. colonies showing yellow colonies on RS agar while, (3) shows Aeromonas spp. on 5% sheep blood agar (5% BSA) exhibiting β-hemolysis zones around the colonies. [file 13104_2021_5716_MOESM6_ESM.pdf]
